# Patterns of religiosity and spirituality of psychiatrists in Brazil and the implications for clinical practice: a latent profile analysis

**DOI:** 10.1186/s12888-020-02929-x

**Published:** 2020-11-23

**Authors:** Maria Cecilia Menegatti-Chequini, Alexandre A. Loch, Frederico C. Leão, Mario F. P. Peres, Homero Vallada

**Affiliations:** 1 Department and Institute of Psychiatry (LIM-21, LIM-23 and ProSER), School of Medicine, University of São Paulo, R. Dr. Ovídio Pires de Campos, 785, Zip Code 05403-010, São Paulo, SP Brazil; 2 Laboratory of Neuroscience (LIM-27), Institute of Psyquiatry, University of São Paulo, São Paulo, SP Brazil; 3 Instituto Nacional de Biomarcadores em Neuropsiquiatria (INBION), Conselho Nacional de Desenvolvimento Científico e Tecnológico, São Paulo, SP Brazil; 4 Departament of Neurology, Albert Einstein Hospital, São Paulo, SP Brazil

**Keywords:** Psychiatrics, Psychiatry, Clinical practice, Religion, Spirituality, Latent profile analysis

## Abstract

**Background:**

Although there is consensus, in psychiatry, over the inclusion of religious and spiritual aspects when evaluating and treating the patient, investigation of these dimensions is rare. There is evidence as to the relationship between psychiatrists’ religious/spiritual beliefs and their willingness to discuss a patient’s religion and spirituality (R/S). Due to the lack of information about how psychiatrists in Brazil deal with R/S in patient care, the aim of the present study is to analyze the religious/spiritual profile of these professionals and to ascertain its influence on attitudes and behavior in clinical practice.

**Methods:**

Five hundred and ninety-two psychiatrists from Brazil answered a questionnaire about R/S in clinical practice. The latent profile analysis was used to search for differences of religious/spiritual profiles. The ANOVA and Pearson’s chi-square tests were employed to identify any correlation between clinical opinion and behaviors according to the different profiles.

**Results:**

Two religious/spiritual profiles were identified (entropy value > 0,96): the so called “less religious” group (*n* = 245), comprised predominantly by men, professionally more experienced, with a higher level of academic education (Master or PhD degrees) and were the ones who least enquired about their patients’ R/S; and the “more religious” psychiatrists (*n* = 347) those who had higher consideration for R/S on health, and who more often addressed R/S with their patients and therefore usually ascribed importance to include R/S in their professional training.

**Conclusion:**

The latent profile analysis produced two distinct classes between the Brazilian psychiatrists according to their R/S views: the more religious professionals, who investigate the patient’s R/S in a more detailed manner, and the less religious, who tend to disregard this aspect.

**Supplementary Information:**

The online version contains supplementary material available at 10.1186/s12888-020-02929-x.

## Background

The importance of addressing religiosity and spirituality (R/S) in clinical practice has been increasingly acknowledged by medical and educational organisations [1]. The American Psychiatric Association (APA) [2] World Psychiatric Association (WPA) [3], Royal College of Psychiatrists (RCP) [4], European Psychiatric Association (EPA) [5] and the Brazilian Association of Psychiatry (ABP) [6] are just a few of the institutions which have verified the need to consider the spiritual dimension in psychiatry education, research and clinical practice. Indeed, the Accreditation Council for Graduate Medical Education (ACGME) [7], which sets out the requirements for the post-graduate medical programs in the USA, stipulated that resident physicians should be capable of demonstrating the skills to deal with the religious and spiritual aspects of patients.

This acknowledgment comes as a result of the abundant evidence of the impact of R/S on physical and mental health [8]. In the last 20 years, a consistent body of research has demonstrated that, although some negative religious/spiritual beliefs may have a harmful effect on health [9, 10], in the majority of cases, religious involvement is associated with positive outcomes in terms of physical health and, principally, mental health [11, 12]. Studies have noted that R/S is associated with a better quality of life [13], lower rates of substance abuse [14], anxiety [15, 16], suicide [17, 18], depression [19, 20] and various other health benefits in general [8, 21] for example it may foster feelings of meaningfulness and peace of mind [22].

The growing awareness about the importance of a new paradigm in healthcare, which views the patient within a comprehensive perspective has resulted in greater interest in physician behavior related to R/S. Researchers from different regions of the world, like the USA, the UK and Denmark, have begun to examine how these professionals deal with R/S in their medical consultations [23, 24]. The results of these studies have shown that, despite the fact the vast majority of physicians, including psychiatrists, agree on the importance of and need to assimilate R/S into clinical work, investigation of the religious/spiritual aspects of patients is rare [25, 26]. R/S is barely considered by physicians, who prefer to delegate the role of discussing matters of this kind to the chaplains [26]. This mismatch may be the result of difficulties encountered by these professionals in the approach to R/S which, generally speaking, relate to lack of time and, mainly, lack of knowledge and training [26, 27].

Moreover, medical practitioners in general, particularly psychiatrists, are less religious than the general population [28, 29]. The difference between the religiosity of mental health professionals and that of their patients has been dubbed “the religiosity gap” [30, 31] and, according to some studies, this more secular characteristic may lead them to ignore the importance of R/S in the clinical setting, thereby undermining the practitioner/patient relationship [32], since patients do feel a need for and expect their practitioners to deal with, religious and spiritual subjects as part of the treatment [33].

There is substantial evidence as to the relationship between physicians’ religious/spiritual beliefs and their willingness to discuss patient R/S [27, 34–36]. A recent study, analyzing over 6000 health professionals, stemmed from 11 studies conducted in 9 countries across 6 continents, and demonstrated that the large differences in moral and religious values among the diverse nations and cultures explains the different clinical approaches of health professionals [23].

In Brazil, we performed two studies with the aim of ascertaining the religious/spiritual profiles of psychiatrists and investigating the field of work of these professionals in terms of the religious/spiritual issues of their patients. The first study [37] was conducted on a sample of psychiatrists from the Brazilian Association of Psychiatry (ABP), representing the population of psychiatrists practicing in Brazil, while the second [38] was carried out using a sample of psychiatrists working in the Institute of Psychiatry at the University of São Paulo, Faculty of Medicine teaching hospital (IPq-HC-FMUSP), reflecting the thoughts and behaviors of the professionals working in one of the most important center for the practice, research and learning of psychiatry in Brazil.

In both studies, the results corroborated previous works [39, 40], noting that the different religious/spiritual characteristics of the psychiatrists were linked to different R/S-related clinical opinions and behaviors. Given these findings, we decided to expand our investigation and analyze both samples jointly from our previous studies in order to identify the different religious/spiritual profiles of Brazilian psychiatrists and investigate how these might impact their attitudes and behaviors, regarding R/S, in psychiatric practice, using the latent profile analysis (LPA) method [41–43]. Investigating the possibility of different patterns of behavior in the approach to R/S might furnish important tools for tailoring suitable training programs for each of these professional’s profiles.

## Methods

This took the form of a quantitative, cross-sectional study, evaluating psychiatrists in Brazil. In the initial phase, 3.120 psychiatrists from the Brazilian Association of Psychiatry (ABP) were selected and invited to complete a questionnaire on R/S and psychiatric practice (a more detailed description can be found in Menegatti-Chequini et al. [37]. In the second phase, 121 psychiatrists were selected, working at the Institute of Psychiatry at the University of São Paulo, Faculty of Medicine teaching hospital (IPq-HC-FMUSP), who were also invited to answer the questionnaire (see detailed description in Menegatti-Chequini et al. [38]. For the present study, samples were gathered from the ABP (484 psychiatrists already analysed in an earlier study [37], plus 35 psychiatrists who responded subsequently) and the IPQ (84 psychiatrists), excluding 11 individuals who were in both samples. The final number of participants in the present study was 592 psychiatrists.

### Measurements

The questionnaire used in this study was developed based on the instrument “Religion and Spirituality in Medicine: Physicians’ Perspectives” developed by Curlin et al. [44], which evaluates the attitudes and behaviors of physicians concerning R/S in clinical practice.

The instrument was adapted for the purposes of this research. It was translated into Portuguese and tested in a pilot study conducted in two phases: initially with 30 health professionals from various specialties and, in the after, with 20 psychiatrists residing at IPq-HC-FMUSP. The questions were considered clear and objective in both steps and no additional adaptation was necessary during the testing phase. The psychiatrists who took part in the pilot study were not included in the final sample.

The questionnaire consisted of self-reporting measurements that accessed three main areas:

- Sociodemographic and professional characteristics

The data included age, gender, marital status, location, degree level, length of professional experience and specialty within psychiatry.

b Participants’ religious/spiritual characteristics

The psychiatrists were questioned about their beliefs in a God or superior power, life after death and reincarnation. There were three response options to each of these questions: yes = 2, undecided = 1, and no = 0. These responses were chosen due to the enormous influence of Christian and spiritualist ideals in the sociocultural scenario in Brazil.

Participants were also polled about their religious affiliations. The answers to this question were divided into five categories: Catholic, Spiritist (including the Afro-Brazilian Spiritism), Protestant or Evangelic, Other religions (encompassing Judaism, Islam, Hinduism, Buddhism, Mormon and Others) and None (including Agnosticism and Atheism). The response options were presented in accordance with the Brazilian context in which Catholics, Evangelists and Spiritists account for the majority of religious devotees [45].

The questionnaire also contained questions relating to the frequency of attendance at churches or temples, the frequency of religious/spiritual practices such as praying, reading religious/spiritual scriptures and spiritual practices, such as meditation and yoga. To all these questions, the response options were: never = 1, once a year or more = 2, once a month or more = 3, once a week or more = 4 and daily = 5.

Measurements of religiosity and spirituality were obtained by applying two questions evaluating the degree to which the participants saw themselves as spiritualized or religious. The questions “To what extent do you consider yourself a religious person?” and “To what extent do you consider yourself a spiritual person?” provided four response options: not at all = 1, slightly = 2, moderately = 3 and very = 4. We did not define the terms religiosity and spirituality, thereby allowing the participants to apply their own interpretations of the concepts. However, they were questioned in such a way as to distinguish between their religiosity and their spirituality.

In order to evaluate coping styles in situations of stress, two questions were applied, extracted from the Spiritual/Religious Coping Scale (RCOPE) [46]; one evaluating religious coping: “ I look to God for strength, support and guidance”, while the other evaluated personal or non-religious coping: “I try to make sense of the situation and decide what to do without relying on God”. Each question has four response options: often = 4, occasionally = 3, rarely = 2 and never = 1.

Intrinsic religiosity, which refers to the degree to which individuals put their religious/spiritual beliefs into practice, was measured by means of two statements that were inspired by the scale from Hoge’s Intrinsic Religious Motivation Scale [47], widely used for these ends: “I try hard to carry my religious/spiritual beliefs over into all my other dealings in life. My whole approach to life is based on my religious/spiritual beliefs”. The response options were as follows: “I completely disagree” = 1, “I moderately disagree” = 2, “I moderately agree” = 3 and “I completely agree” = 4.

c Opinions and behaviors related to R/S and the approach to R/E in clinical practice and in professional training.

With regard to the influence R/S may have on the actions of the medical practitioner, psychiatrists responded to questions evaluating: whether their own beliefs influenced their clinical work; whether they saw medicine as a calling or a mission; whether experience as a medical professional led them to question their own beliefs and, whether they considered it a challenge to remain faithful to their beliefs working as a medical professional. To all these questions, the response options were as follows: strongly = 4, moderately = 3, slightly = 2 and not at all = 1.

The participants also responded if they considered it important to include R/S into clinical practice, medical training and continuing medical education. The questions had four possible responses: very important = 4, reasonably important = 3, a little important = 2 and not important = 1.

With respect to opinions on the influence of R/S on health, the psychiatrists answered questions about the degree to which they considered R/S might influence patients’ decisions concerning the indicated treatment and if R/S might affect wellbeing and the clinical evolution of their patients. The response options were: frequently = 4, occasionally = 3, rarely = 2 and never = 1.

As for the approach to R/S in clinical assessments, the participants were questioned as to the frequency with which they would gather their patients’ R/S histories and if they felt that evaluating a patient’s R/S was something the medical professional should or could do. To the first question, the response options were: frequently = 4, occasionally = 3, rarely = 2 and never = 1, while for the second question, the responses were no = 1, yes = 2 and undecided = 3.

To identify the barriers and difficulties encountered by the participants in addressing religious/spiritual issues with their patients, they were asked to answer a multiple-choice question with the following alternatives: 1) None, 2) Fear of exceeding the role of a doctor, 3) Lack of training, 4) Lack of time, 5) Not being comfortable with the issue, 6) The religious/spiritual aspect is not relevant for the patient, 7) Fear of offending the patient, 8) Fear that peers may not approve, 9) It is not the doctor’s job, 10) Do not know why.

### Statistical analysis

The sample’s continuous and categorical data were described. For the LPA, the sample of psychiatrists from the IPq-HC-FMUSP and from the ABP was viewed as a single sample.

LPA is a statistical method used to identify homogeneous groups or classes using categorical and continuous multivariate data [41]. It is designed to verify whether the data can or cannot be grouped according to similar response patterns. LPA is similar to latent class analysis (LCA). While the former may handle continuous and categorical variables, the latter only deals with ​​dichotomous variables. The analysis is performed by attempting to pigeonhole the data in a specific number of categories and observing which parameters this categorization generates. The optimal solution/categorization will be chosen according to which parameters are generated. The following parameters were employed in this study: a) Akaike information criterion (AIC), where the lowest value indicates the best quality of information supplied by the categories generated, b) sample-size adjusted Bayesian information criterion (BIC), where the lowest value also indicates the best quality of information supplied by the categories generated, c) entropy, a homogeneity measure of the classes generated, ranging from 0 to 1, in which 1 denotes perfectly homogeneous classes, d) Parametric bootstrapped likelihood ratio test, in which *p* < 0.05 indicates there is no difference between classifying the data into *n* categories or into *n-1* categories, and e) the Lo-Mendell-Rubin (LMR) adjusted likelihood ratio test, where p < 0.05 indicates that the real solution is statistically different from the solution with *n-1* classes, indicating that the real solution should be chosen over the solution with *n-1* classes. There is no perfect solution for the division of data into profiles, so, according to Muthen & Muthen [42], the best solution must be presented taking into account the above parameters and clinical judgment.

The analyses were conducted using Mplus Version 8 for Mac OS [48].

## Results

A total of 592 psychiatrists took part in this study. In this sample, the mean age was 47.96 (SD = 11.7), 60% were men and more than two-thirds were married. As far as professional experience is concerned, the data showed that the average length of time working in psychiatry was 20.63 years (SD = 11.65) and, in terms of level of education, the majority of the sample had a post-graduate qualifification and had earned a speciality in Psychiatry. As for religious affiliation, 66.5% of the psychiatrists stated at least one religion, the majority being Catholic (Table 1).

**Table 1:** Sociodemographic and professional characteristics of the study sample (*n* = 592)

	*n* (%)
Age (years; mean, SD)	47.96 (11.66)
Sex (female; %, *n*)	237 (40.0)
Civil status (married; %, *n*)	414 (70.8)
Educacional degree	
Residency	153 (26.1)
Specialization	204 (34.8)
Masters	101 (17.2)
Doctorate^a^	129 (22.0)
Missing values	5
Years as a psychiatrist (years; mean, SD)	20,63 (11.65)
Religious affiliation (%, *n*)	
Catholic	186 (31.5)
Protestant or Evangelical	40 (6.8)
Spiritist	104 (17.6)
Other religion	63 (10.6)
None	198 (33.5)
Missing value	1

^a^Includes the categories “Post-doctorate” and “Associate professors”

With regard to the participants’ opinions and behaviors in respect to R/S in health and clinical practice, the results showed that 44.6% considered that R/S frequently interferes with the patient’s decision concerning the indicated treatment; the remainder of the respondents were divided between the following responses: occasionally (37.7%), rarely (13.6%) and never (4.1%). As for the consideration that R/S affects patients’ wellbeing, 64.8% responded frequently, 29.6% occasionally, 3.8% rarely and 1.8% never. To the question evaluating whether they felt it important to include R/S in medical training, they answered as follows: 43.2% very important, 28.9% reasonably important, 14.9% a little important and 12.9% not important. The inclusion of R/S in psychiatric training was considered very important by 40.6, 30.1% responded that it was reasonably important, 16.7% a little important and 12.6% not important. The data also showed that 46.5% of psychiatrists frequently broached the religious and spiritual issues of their patients, 33.8% occasionally, 14.6% rarely with just 5.1% saying they never addressed R/S in clinical practice.

Table 2 shows the latent profile analysis (LPA) of the psychiatrists’ responses to the questionnaire. The results indicated that the best LPA output was with two classes of psychiatrists, as this presented the best entropy value and was the number of classes that had a statistically significant Lo-Mendell-Rubin value.

**Table 2:** Latent profile analysis of psychiatrists’ answers to the questionnaire on Religion /Spirituality in psychiatric practice

	AIC	Adjusted BIC	Entropy	Parametric bootstrapped LRT	*p*	Lo-Mendell-Rubin Adjusted LRT Test
2 classes	23,291.237	23,354.096	**0.967**	−13,756.570	0.0000	**0.0000**
3 classes	22,306.700	22,393.735	0.927	−11,593.618	0.0000	0.4218
4 classes	21,858.771	21,969.983	0.925	−11,081.349	0.0000	0.7613
5 classes	21,724.447	21,859.835	0.933	−10,837.386	0.0000	0.7357

**Fig. 1:**
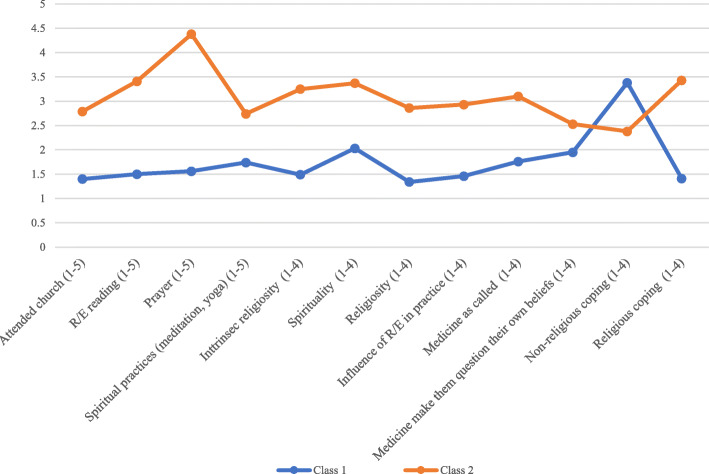
Psychiatrists’ religious/spiritual characteristics according to different religious profiles

Figure 1 depicts the religious/spiritual characteristics of the psychiatrists and their opinions related to R / S in clinical practice, according to the different profiles. Profile 1 we call the “less religious” as, when comparing these individuals to the psychiatrists in the other profile, they were the ones who least frequently attended religious/spiritual places of worship (mean of 1.4 vs. 2.79), who least read religious/spiritual scriptures (mean of 1.5 vs. 3.41), who prayed less (mean of 1.56 vs. 4.38) and who demonstrated the lowest frequency of spiritual practices, such as meditation and yoga (mean 1.74 vs. 2.74). They also exhibited lower mean values for intrinsic religiosity (1.49 vs. 3.25), spirituality (mean 2.03 vs. 3.37), religiosity (mean 1.34 vs. 2.86) and religious coping (mean 1.41 vs. 3.43). For the sake of consistency, and solely for the question that evaluated non-religious coping, the rates were higher (mean 3.38 vs. 2.38). As for R/S-related opinions in clinical practice, they were the ones who least considered that their religious/spiritual beliefs influenced their work as medical professionals (mean 1.46 vs. 2.93), who least believed that medicine was a calling or mission (mean 1.76 vs. 3.10) and, moreover, who least considered that medicine practice might make them question their own beliefs (mean 1.95 vs. 2.53). All the above reported results presented *p* < 0.001.

**Fig. 2:**
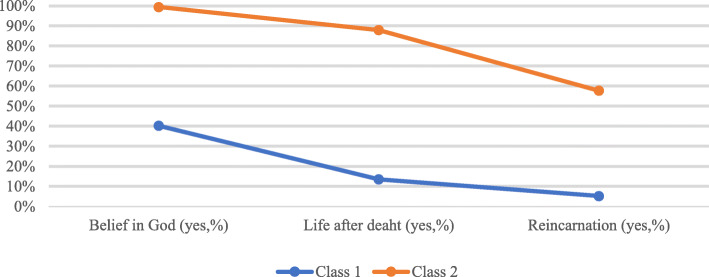
Psychiatrists’ religious/spiritual beliefs according to different religious profiles

Figure 2 shows the religious/spiritual beliefs of the psychiatrists according to the different profiles. We can see that profile 1 also groups individuals with the lowest percentages of believing in God (40.2% vs. 99.4%), life after death (13.5% vs. 87.9%) and reincarnation (5.2% vs. 57.7%). These results reached also *p* < 0.001.

As demonstrated in Figs. 1 and 2, profile 2 groups the most “believers” psychiatrists, ie those who had the highest indexes of religious/spiritual beliefs and practices and was therefore named as “more religious”.

Table 3 describes the conditional likelihood of each response item in the respective profile. Profile 1 consisted of 245 (41.5%) psychiatrists with an average age of 48.54 while profile 2 accommodates the majority of the participants (*n* = 347, 59.6%) with an average age of 47.54.

**Table 3:** Sample characteristics according to different religious profiles

	Class 1 (*n* = 245) Less religious	Class 2 (*n* = 347) More religious	p
*Sociodemographics and professional data*			
Age (years; mean, SD)	48.54 (11.95)	47.54 (11.46)	0.313^b^
Sex (female; %, n)	33.5% (82)	44.7% (155)	**0.006**^**a**^
Civil status (married; %, n)	73.4% (177)	68.7% (237)	0.473^a^
Education (Phd + post-doc; %, n)	28.0% (68)	17.7% (61)	**0.001**^**a**^
Years as a psychiatrist (years; mean, SD)	21.79 (11.75)	19.82 (11.53)	**0.044**^**b**^
*Religious characteristics*			
Religious affiliation (none; %,n)	69.3% (169)	8.4% (29)	**0.000**^**a**^
R/S medical formation (mean, SD)	2.47 (1.14)	3.37 (0.81)	**0.000**^**b**^
R/S psychiatrist formation (mean, SD)	2.39 (1.09)	3.36 (0.80)	**0.000**^**b**^
R/S affects patient decision (mean, SD)	3.01 (0.87)	3.36 (0.78)	**0.000**^**b**^
R/S affects patient’s well-being (mean, S D)	3.29 (0.78)	3.75 (0.47)	**0.000**^**b**^
R/S approach (mean, SD)	3.06 (0.97)	3.33 (0.78)	**0.000**^**b**^

^a^Chi-square test

^b^ANOVA test

Bold**:** significant correlations

Comparing individuals in the two profiles, we can see that the profile 1 psychiatrists who had been practicing psychiatry longer (an average of 21.79 years vs. 19.82; p < 0.05), was the group which contained more males (66.5% vs. 55.3%; *p* < 0.01) and embraced psychiatrists of a higher educational level: 28.0% had a Doctorate, as a minimum, and some also had postdoctoral degrees or were associate professors, while in profile 2, the percentage was just 17.7%, (*p* = 0.001).

The majority (69.3%) of individuals in profile 1 stated they did not observe any religion, unlike profile 2 where 91.6% of psychiatrists indicated a religious affiliation (*p* < 0.001). With regard to the opinions related to R/S and health, the psychiatrists in profile 1 were the ones who least considered that R/S exerted an influence on patients’ decisions about the indicated treatment (mean = 3.01 vs. 3.36) and that R/S could affect the patient’s health and wellbeing (mean = 3.29 vs. 3.75). The individuals in profile 1 were also those who considered least important the inclusion of R/S in medical training (mean = 2.47 vs. 3.37) or psychiatric training (mean = 2.39 vs. 3.36). As for the R/S approach in clinical practice, we found that the psychiatrists in profile 1 were those who least asked about the religious/spiritual issues of their patients (mean = 3.06 vs. 3.33). All these results also presented *p* < 0.001.

## Discussion

The results from this sample indicated that Brazilian psychiatrists are split into two different religious/spiritual profiles. The “less religious” profile was the smaller group, consisting of those psychiatrists with lower indices of belief and frequency of religious/spiritual practices; more men than women belonged to this profile, they were the participants with the highest educational levels, who had been working for longer in the field of psychiatry and who least broached R/S in clinical practice. The “more religious” profile consisted of psychiatrists who were more devout and active in religious life; this was the group that most took into account the influence of R/S within healthcare and medical treatment, that ascribed greater importance to the inclusion of R/S in professional training and which most addressed the R/S of patients.

The psychiatrists within the “more religious” profile were designated as such due to their higher indices of religiosity, spirituality, intrinsic religiosity, religious coping, belief in God, life after death and reincarnation and, logically, were the ones who were the most dedicated to religious and spiritual practices, such as attending religious services, praying and reading religious/spiritual scriptures. They were also the ones who most felt their beliefs had an influence on their clinical activity, and that these practices influenced their personal beliefs. These results are similar to those found in a previous study in which a potential reciprocal influence of R/S is discussed regarding clinical practice and its influence on the professional’s religiosity [49]. They also match the findings of a meta-analysis of ten samples of professionals from seven countries, which showed very strong positive correlation between the professional’s degree of religiosity and the perception of the effects it had on his/her work [50].

Another characteristic that defined the “more religious” profile was considering medicine as a calling, this being compatible with data found in other studies in which medical professionals in general and psychiatrists with higher levels of R/S have a greater tendency to regard the practice of medicine as a vocation [51, 52].

The majority of psychiatrists belonging to the “less religious” profile were male, showing, as it does in the literature, that female psychiatrists attribute greater importance to R/S in their lives [53, 54]. In addition to grouping a larger number of men, the “less religious” profile, not surprisingly, also brought together psychiatrists with less religious affiliation, data similar to those found in the Brazilian population in general, in which the male sex leads the “no religion” group, with a proportion of 9.7% for men against 6.4% for women [45]. A large number of empirical studies have demonstrated that religiosity in women is greater than in men, this difference having sparked intense investigation since as far back as 1930 [55, 56]. However, although many theories attempt to explain the reasons for this difference, ranging from approaches of a sociological [57] and psychological [58] nature to those that substantiate men’s greater non-religiosity on physiological grounds [59], no conclusive empirical studies yet exist capable of justifying any concrete position on the matter. So far, what can be seen is an inchoate consensus in terms of the multifactorial nature of its origins [60].

The psychiatrists who had worked for a longer time within the field were also more concentrated in the “less religious” profile and, in this regard, we need to pay attention to the possibility that these data represent more of a generational phenomenon than a fact necessarily relating to the length of time within the field, as has already been discussed in one of our studies, in which longer-serving psychiatrists were shown to have less religious affiliation [37].

In this regard, we must consider that, at the beginning of the twentieth century, some theories, such as those of Sigmund Freud [61, 62], ascribed a neurotic character to religious experiences, guiding much of the theoretical currents in psychiatry and psychology which began to pathologize religious/spiritual beliefs and practices [63].

Even in the seventies, mystical manifestations or experiences were still almost exclusively associated with pathological behaviors of the psychotic type, or with a phenomenon of regression or flight, as proclaimed in 1976 by the GAP’s (Group for the Advancement of Psychiatry) “Committee on Psychiatry and Religion”, in the USA [64].

Although this negative perception of religious experiences was not based on scientific research or systematic studies, merely derived from clinical observation and, mainly, the personal opinions and beliefs of authors [65], its persistence heavily influenced the field of study that examines the intersection of science and religion [66] and ended up sharply reflecting on the development of mental health professionals. The reflection of this influence is probably more conspicuous in professionals that were practicing at the time these ideas predominated.

Moreover, in the beginning, psychiatric approaches were partisan; some theories supported the predominance of biological aspects in the etiology of mental illness, while others pointed to psychological factors as their determining cause. Only recently have more comprehensive models emerged, which incorporate diverse elements in the understanding of mental health [67]. Nowadays, psychiatry aims towards an integral approach and takes into consideration the interaction of biological, psychological, social, and religious/spiritual factors in medical assessments and interventions [68].

Regarding the influence of R/S on the health and treatment of patients, the results found here indicate that the “more religious” participants were those that most believed in this kind of impact. These data are similar to those found in the literature, showing that psychiatrists with a higher index of intrinsic religiosity had a greater perception of the influence of R/S on the health of patients and, the more religious they were, the more they tended to regard this influence as positive [27, 69]. Other studies with health professionals from several specialties also signaled this tendency [26, 70].

The “less religious” psychiatrists were those that least discussed R/S with their patients, underlining the “religiosity gap*”* phenomenon between doctors and patients, so widespread in the literature [31, 32, 71], in which doctors, by not attaching importance to R/S in their own lives, assume that the same is true of their patients and end up disregarding these aspects, already understood to be important for the majority of them [33].

Several studies have demonstrated the relationship which exists between medical professionals’ religious/spiritual beliefs and their tendencies to approach (or not) R/S in clinical care [36, 39, 72]. Generally, practitioners with greater self-perception of R/S exhibit a greater tendency to investigate patients’ R/S [37, 54], the same holding true for those that say they indulge more frequently in public and private religious practices [27, 34], unlike the health professionals that do not identify with any religion and do not take part in religious gatherings, who are less inclined to empathize with the R/S of patients and/or to recommend them for spiritual counseling [73].

The analyses performed also show that psychiatrists in the “less religious” profile regard the inclusion of R/S in professional training as of little importance, which appears to be consistent for professionals who accord little importance to the influence of R/S on health and are less willing to address patients’ R/S, differently from the study of Rensburg et al. [74], in which all academic psychiatrists agreed that spirituality should be assimilated into clinical assessment and training in psychiatry.

One of the most relevant results of this study is in respect of education. Psychiatrists belonging to the “less religious” profile had the highest levels of education: it was the group with the most PhDs, Post-doctorates and associate professors. This negative relationship between religion and education had already been verified in previous studies [75, 76], but there is no consensus on the matter, as studies exist that counter this idea, showing a positive association between religious beliefs and education [77–79]. There is, moreover, evidence that this negative relationship has diminished significantly over recent years [80], suggesting the plausibility of religion and science being compatible visions of the world [81].

However, our data seem to corroborate the erstwhile theory that education leads to a decline in religion [82], which might be understood as part of the process of the desacralization or secularization of society, where the fundaments of religion were replaced by belief in science, secular beliefs, generally opposed to religious beliefs [83, 84].

Again, we are faced with the possibility of our data representing a generational phenomenon, as obtaining academic titles demands more time in the field and, therefore, more exposure to anti-religious theories which may have influenced the beliefs of these psychiatrists.

On the other hand, being more educated and highly trained professionals, it might be expected they would be more conscious of the importance of evidence-based medical practice and, therefore, that they would approach these topics without prejudice and without regard to their personal religious values, since there currently exists abundant proof of the impact of R/S on mental health. However, this is not what our data showed as those psychiatrists with a “less religious” profile, in addition to ascribing little importance to R/S in the patient’s health and treatment, were also those who least addressed the topic in clinical practice. Considering the influence of R/S as negative for patients is itself sufficient reason to include it as part of the medical treatment instead of ignoring it [85]. Ascertaining if R/S plays any role in the illness and if this influence is positive or negative for the patient’s recovery, is fundamental in avoiding diagnostic error [86].

It is necessary to understand that psychiatry does not lose its scientific character when investigating the relationship of R/S in healthcare, as they are phenomena that can be scientifically investigated [87].

The latent profile analysis in this study identified polarized patterns in the clinical practice of Brazilian psychiatrists related to their personal beliefs; the ones who were more religious explored R/S, while the less religious tended to ignore it.

These findings suggest that psychiatrists (both religious and non-religious) must, therefore, undergo training regarding the importance of R/S to the causes, diagnoses, and, treatment of mental disorders [88]. Moreover, this training must be able to expand these professionals’ awareness of the possible evaluative biases in the assessment of their patients’ R/S. It must inform them on the relevance of “good psychiatric practice”, which observes important limits with regard to the approach to R/S and rules out the possibility of any form of proselytization, whether it be on account of religious, political or atheistic beliefs [89]. It is fundamental for psychiatrists to contemplate their own religious values and to respect ethical limits in their work, prioritizing comprehensive care for the patient.

Thus, training programs must be directed towards all psychiatrists, including academic professionals with the highest levels of education, as it is precisely these professionals that will be educating future generations of psychiatrists, in order to avoid the potential perpetuation of neglect when addressing R/S.

### Limitations

The present study does have some methodological limitations, perhaps the most important of which is the fact that it is a cross-sectional study that does not allow for formal inferences regarding causality. Furthermore, data was obtained via self-reports from psychiatrists, a form of measurement that is more susceptible to biases.

Another probable limitation of the sample is the possibility of the topic having aroused more interest, in both very religious and very non-religious individuals, to participate. In a similar study previously performed by Curlin et al. [39], the non-religious medical professionals seem to have been more willing to answer the questionnaire.

As there is still no consensus in the literature regarding the concepts of religiosity and spirituality, we allowed the psychiatrists to use their own definitions to answer the questions that drew a distinction between the terms. Thus, interpretation of results and their comparison with other studies must be conducted with caution.

Lastly, future investigations should include the aspects psychiatrists consider positive or negative in the questions that evaluate their perception of the influence of R/S on the health and treatment of patients; furthermore, it would be important to find out about their conceptions of R/S, how they understand and handle the approach to R/S and if they have undergone training in the area.

## Conclusions

The results of this study contribute important data to the literature of the area. They show that, if we consider the R/S characteristics of psychiatrists in Brazil, we have basically two distinct types of professionals. Comparing the two groups, we concluded that those psychiatrists who were seen to be less religious/spiritual, have been in psychiatry practice for a longer period of time, are more highly educated and are the ones who tend to lend scant importance to religious/spiritual aspects of their patients. On the other hand, psychiatrists with greater levels of R/S, although they may have fewer academic qualifications and less experience in the field of psychiatry, are the professionals that attribute greater relevance to the religious/spiritual aspects when assessing and treating their patients.

## Supplementary Information

**Additional file 1.** Questionnaire.

## Data Availability

The dataset is available upon request (contact the corresponding author).

## Abbreviations

R/S: Religiosity and spirituality
APA: American Psychiatric Association
WPA: World Psychiatric Association
RCP: Royal College of Psychiatrists London
EPA: European Psychiatric Association
ABP: Brazilian Association of Psychiatry
ACGME: Accreditation Council for Graduate Medical Education
IPq-HC-FMUSP: Instituto de Psiquiatria do Hospital das Clinicas da Faculdade de Medicina da Universidade de Sao Paulo
LPA: Latente profile analysis
RCOPE: Spiritual/religious coping scale
AIC: Akaike information criterion
BIC: Bayesian information criterion
LMR: Lo-Mendell-Rubin
GAP: Group for the advancement of psychiatry
