# Supplementary material for: Patterns of religiosity and spirituality of psychiatrists in Brazil and the implications for clinical practice: a latent profile analysis
Source: BMC Psychiatry. 2020 Nov 23;20:546. doi: 10.1186/s12888-020-02929-x (PMC7682088; doi:10.1186/s12888-020-02929-x)
Supplement: Supplementary file 1 — Additional file 1. Questionnaire. [file 12888_2020_2929_MOESM1_ESM.docx]

**QUESTIONNAIRE**

**Spirituality and Religion in Psychiatric Practice**

| **I AGREE TO TAKE PART IN THE INTERVIEW.** |
| --- |
| 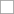 **YES**    **1. Age **  **2. Adress:**  **City **  **State **  **3. Gender**  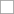 ^1^ Female  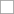 ^2^ Male  **4. Marital status**  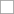 ^1^ Married/Living with a partner  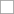 ^2^ Single  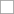 ^3^ Divorced  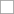 ^4^ Widowed |
| **5. Level instruction** |
| 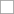 ^1^ Undergraduate Degree |
| 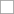 ^2^ Residency |
| 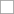 ^3^ Specialization |
| 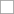 ^4^ Masters |
| 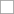 ^5^ Doctorate |
| 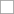 ^6^ Post-doctorate |
|  |
| **6. How long ago did you finish your residency? ** |
| **7. In which areas of psychiatry do you work? (Subespeciality)**  **(Check all that apply)** |
| 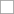 Adult |
| 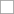 Child |
| 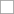 Old age |
| 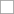 Forensic |
| 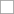 Others (specify) |
| **8. How long have you been working in psychiatry? ** |
| **9. What is your religious affiliation?** |
| 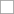 ^1^ Catholic |
| 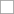 ^2^ Protestant  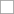 ^3^ Evangelical  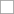 ^4^ Kardecian Spiritist |
| 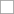 ^5^ Afro-Brazilian Spiritism  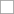 ^6^ Jewish  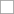 ^8^ Muslim  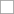 ^9^ Buddhist  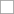 ^11^ Agnostic  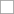 ^12^ Atheist  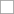 ^13^ None  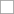 ^14^ Others (specify) |
|  |
| **10. How often do you attend religious services?**  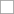 ^1^ Never,  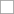 ^2^ Once a year or more,  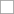 ^3^ Once a month or more,  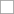 ^4^ Once a week or more  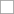 ^5^ Daily |
| **11. How often do you do some form of religious/spiritual reading?** |
| 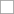 ^1^ Never, |
| 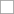 ^2^ Once a year or more, |
| 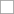 ^3^ Once a month or more, |
| 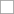 ^4^ Once a week or more |
| 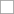 ^5^ Daily |
|  |
| **12. How often do you engage in some form of prayer?** |
| 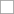 ^1^ Never, |
| 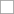 ^2^ Once a year or more, |
| 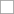 ^3^ Once a month or more, |
| 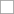 ^4^ Once a week or more |
| 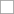 ^5^ Daily |
|  |
| **13. How often do you perform some form of non-religious, spiritual practice (such as meditation, yoga, etc.)?** |
| 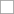 ^1^ Never, |
| 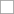 ^2^ Once a year or more, |
| 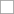 ^3^ Once a month or more, |
| 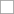 ^4^ Once a week or more |
| 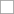 ^5^ Daily |
|  |
| **14. Do you believe in God or a superior power?** |
| 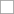 ^0^ No |
| 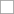 ^1^ Undecided  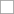 ^2^ Yes  **15. Do you believe in life after death?**  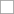 ^0^ No  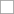 ^1^ Undecided  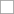 ^2^ Yes |
| **16. Do you believe in reincarnation?** |
| 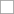 ^0^ No |
| 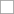 ^1^ Undecided |
| 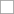 ^2^ Yes  **About your intrinsic religiosity**  *Intrinsic religiosity refers to how much a person’s religion is the main guiding motivation that gives meaning to his/her life.* |
| **17. I try hard to carry my religious/spiritual beliefs over into all my other dealings in life. My whole approach to life is based on my religious/spiritual beliefs.**  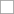 ^1^ I completely disagree  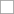 ^2^ I moderately disagree  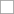 ^3^ I moderately agree  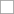 ^4^ I completely agree |
| **18. To what extent do you consider yourself a spiritual person?**  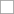 ^1^ Not spiritual at all  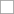 ^2^ Slightly spiritual  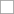 ^3^ Moderately spiritual  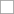 ^4^ Very spiritual |
| **19. To what extent do you consider yourself a religious person?** |
| 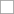 ^1^ Not religious at all |
| 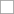 ^2^ Slightly religious |
| 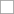 ^3^ Moderately religious |
| 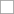 ^4^ Very religious |
|  |
| **20. My religious beliefs influence my practice of medicine.** |
| 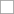 ^1^ Not at all  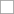 ^2^ Slightly |
| 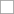 ^3^ Moderately |
| 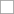 ^4^ Strongly |
| **21. For me, the practice of medicine is a calling** |
| 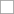 ^1^ Not at all  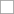 ^2^ Slightly |
| 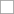 ^3^ Moderately |
| 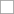 ^4^ Strongly  **22. My experiences as a physician have caused me to question my religious beliefs.** |
| 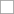 ^1^ Not at all  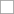 ^2^ Slightly |
| 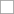 ^3^ Moderately |
| 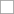 ^4^ Strongly  **23. I find it challenging to remain faithful to my religion in my work as a physician.**  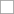 ^1^ Not at all  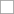 ^2^ Slightly  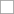 ^3^ Moderately  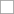 ^4^ Strongly  **Religious and spiritual coping**  *Reply to the questions listed below with the option that best expresses the way you cope with situations of adversity in your life.*  **24. I try to make sense of the situation and decide what to do without relying on God.**  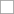 ^1^ Never  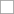 ^2^ Rarely  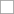 ^3^ Occasionally  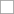 ^4^ Often  **25. I look for a God as a source of strength, support, and guidance.**  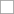 ^1^ Never  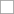 ^2^ Rarely  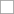 ^3^ Occasionally  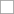 ^4^ Often  **26. Do you consider it important to integrate patients’ religion/spirituality in clinical practice?**  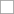 ^1^ Not important  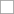 ^2^ Little important  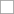 ^3^ Reasonably important  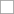 ^4^ Very important |
| **27. Do you consider it important that the issues of religion/spirituality are including in medical training?**  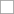 ^1^ Not important  ^2^ Little important  ^3^ Reasonably important  ^4^ Very important  **28. Do you consider it is important for the topic of R/S to be included in continuing psychiatric education?**  ^1^ Not important  ^2^ Little important  ^3^ Reasonably important  ^4^ Very important |
|  |
| **29. In your opinion, how often do religious/spiritual beliefs affect patients’ decisions concerning the recommended treatment?** |
| ^1^ Never |
| ^2^ Rarely |
| ^3^ Occasionally |
| ^4^ Often |
|  |
| **30. In your opinion, how often do religious/spiritual beliefs affect a patient’s wellbeing and clinical course?** |
| ^1^ Never |
| ^2^ Rarely |
| ^3^ Occasionally |
| ^4^ Often |
|  |
| **31. Enquiring about the religion/ spirituality of the patient is something the psychiatrist can (or should) do.** |
| ^1^ No |
| ^2^ Yes |
| ^3^ Undecided |
| **32. How often do you enquire about patients’ religious/spiritual issues?** |
| ^1^ Never |
| ^2^ Rarely |
| ^3^ Occasionally |
| ^4^ Often |
|  |
| **33. Challenges or barriers to approaching patients’ religion/spirituality.**  **(Check all apply)**  None  Fear of exceeding the role of a doctor  Lack of training  Lack of time  Not being comfortable with the issue  The religious/spiritual aspects it not relevant for the patient  Fear of offending the patient  Fear that peers may not approve  It is not the doctor’s job  Do not know why |

**If you wish to make a comment, opinion or suggestion, please use the space below. They will be very useful for this study.**

**THANK YOU FOR YOUR PARTICIPATING!**
